# Comparison of Oil-Seed Shell Biomass-Based Biochar for the Removal of Anionic Dyes—Characterization and Adsorption Efficiency Studies

**DOI:** 10.3390/plants13060820

**Published:** 2024-03-13

**Authors:** Shrikanta Sutradhar, Arijit Mondal, Felix Kuehne, Oliver Krueger, Sudip K. Rakshit, Kang Kang

**Affiliations:** 1 Biorefining Research Institute (BRI), Chemical Engineering Department, Lakehead University, 955 Oliver Road, Thunder Bay, ON P7B 5E1, Canada; ssutradh@lakeheadu.ca; 2 Department of Biological Science, Indian Institute of Science Education and Research (IISER), Kolkata 741 246, India; am19ms001@iiserkol.ac.in; 3 Berliner Hochschule für Technik BHT, Luxemburger Straße 10, 13353 Berlin, Germany; fkhne@lakeheadu.ca (F.K.); oliver.krueger@bht-berlin.de (O.K.)

**Keywords:** oil-seed shell biomass, biochar, dyes removal, adsorbent, adsorption mechanism

## Abstract

This research investigated the synthesis of biochar through the direct pyrolysis of pre-roasted sunflower seed shells (SFS) and peanut shells (PNS) and compared their application for the effective removal of textile dyes from wastewater. Biochar prepared at 900 °C (SFS900 and PNS900) showed the highest adsorption capacity, which can be attributed to the presence of higher nitrogen content and graphite-like structures. CHNS analysis revealed that PNS900 exhibited an 11.4% higher carbon content than SFS900, which enhanced the environmental stability of PNS biochar. Fourier-transform infrared spectroscopy (FTIR) and X-ray diffraction (XRD) analyses of the produced biochar indicated the degradation of cellulosic and lignin moieties. X-ray photoelectron spectroscopy (XPS) revealed a 13.8% and 22.6% increase in C-C/C=C mass concentrations in the SFS900 and PNS900, respectively, and could be attributed to the condensation of polyaromatic structures. Batch experiments for dye removal demonstrated that irrespective of dye species, PNS900 exhibited superior dye removal efficiency compared to SFS900 at similar dosages. In addition to H-bonding and electrostatic interactions, the presence of pyridinic-N and graphitic-N can play a vital role in enhancing Lewis acid-base and π-π EDA interactions. The results can provide valuable insights into the biochar–dye interaction mechanisms.

## 1. Introduction

Water decontamination is a critical topic in environmental science and public health. Contaminants in water sources, such as heavy metals, organic pollutants, and emerging contaminants like pharmaceuticals and personal care products, pose significant risks to both human health and the environment [1]. Water treatment methods, such as coagulation, flocculation biodegradation, catalytic oxidation, and membrane filtration, each have their advantages and drawbacks [2]. While coagulation, flocculation, and catalytic oxidation are cost-effective, they require high volumes of chemicals. Membrane filtration is efficient but expensive [2,3]. Nanofiltration is the advanced technology of membrane filtration, which may not be suitable for practical application in textile wastewater treatment due to dense separation layers [2]. Adsorption, however, is an economical and effective technique for removing wastewater pollution. Its operational simplicity and potential for regeneration make it attractive [4]. Adsorption encompasses three main types: physisorption (interaction between the adsorbent and adsorbate), chemisorption (interaction between the adsorbent and solvent), and electrostatic interactions (interaction between the adsorbate and solvent) [4,5,6]. Researchers have delved into the underlying mechanisms occurring on the surface of the adsorption material. Researchers have explored the underlying mechanisms, and factors like ion concentration, dosage, temperature, pH, time, and stirring speed impact adsorbent efficiency [4,6].

Biochar (BC) is a carbon-rich material produced through the process of pyrolysis involving organic materials like agricultural residues, wood, or other biomass resources. During pyrolysis, organic matter is subjected to elevated temperatures in an oxygen-limited environment, leading to the breakdown of volatile compounds and the formation of durable, porous carbon structures [7,8,9]. This porous structure grants biochar a significant surface area and provides numerous chemically active sites for the adsorption of contaminants [10]. This property enables it to effectively eliminate a broad spectrum of pollutants from both water and air. Furthermore, biochar can be customized and adjusted to augment its adsorption capacity for specific contaminants. Introducing surface modifications and incorporating additional materials can enhance its performance to suit precise applications. Another noteworthy aspect of biochar is its stability and long-lasting nature, primarily attributable to its high carbon content. Research indicates that biochar with a hydrogen-to-carbon (H/C) ratio of less than or equal to 0.7 and an oxygen-to-carbon (O/C) ratio of approximately 0.4 demonstrates remarkable stability and can persist in soil for well over a millennium [11,12].

Worldwide, 49.6 million MT of sunflower seeds [13] and 48 million MT of peanuts [14] are produced, which generates 50 and 25% of their weight as solid wastes (mostly oil-seed shells). These oil-seed shells are mainly in the form of lignocellulosic biomass and could be utilized for various potential applications [13]. Commercially, these oil seed shells are burnt as a source of fuel, litter for poultry, animal food, and additives in fertilizer [13,15,16]. However, these lignocellulosic biomasses can be pyrolyzed to produce higher-value products like biochar for efficient removal of organic pollutants such as textile dyes from wastewater. The removal of textile dyes, including Methylene Blue [17,18], Remazol Brilliant Blue (RBB) [19,20], and Congo Red [21,22] (CR) from wastewater are efficiently achieved through biochar. Biochar, derived from organic waste, acts as an effective adsorbent, capturing and immobilizing dye molecules in its porous structure. During pyrolysis, biomass undergoes distinct transformations at various temperature ranges. Biochar produced at lower temperatures (300–400 °C) exhibits a more diversified organic character attributed to aliphatic and cellulose-type structures [23,24]. At temperatures up to 480 °C, the biochar retains acidic oxygenated functional groups like phenolic acid and carboxyl, which is more effective for cation exchanges [24]. Biochars obtained at temperatures exceeding 600 °C and more, resulting in higher specific surface areas, demonstrate increased surface microporosity and are more stable and hydrophobic [24,25,26]. Structurally, lower temperatures result in biochar with more functional groups such as carbonyl (-C=O), carboxylic (-COOH), and hydroxylic (-OH) groups, offering higher surface reactivity. As temperatures rise, these groups decrease, yielding a more stable, carbon-rich structure. Tailoring pyrolysis conditions allows for versatile applications, impacting biochar’s physical and chemical properties for specific purposes, such as soil improvement and carbon sequestration. As per our literature search, no recent study demonstrated the direct pyrolysis of sunflower seed shells (SFS) and peanut seed shells (PNS) at higher temperatures or performed comparative adsorption studies of RBB and CR dye removal from wastewater.

The primary novelty of the current work includes the simplified process of direct pyrolysis conversions of two novel biomass resources (i.e., SFS and PNS) into value-added biochar and their good adsorption efficiency on RBB and CR textile dyes. Also, this study is original in providing a detailed comparison and in-depth discussions on the biochar characteristics that might impact dye removal mechanisms.

## 2. Results and Discussion

### 2.1. Effects of Raw Materials and Temperatures on Biochar Yield and Quality

This study demonstrates the efficient BC production from SFS and PNS at varying temperatures, as shown in Table 1. It can be noticed that increasing the pyrolysis temperature decreased the yield of BC from 49.2% to 30.2% and 56.1 to 30.2% for SFS and PNS, respectively. The thermal decomposition of the lignocellulosic biomass from earlier studies showed that decreasing BC yield can be directly related to the removal of the volatile matter (<170 °C), degradation of the hemicellulose (220–350 °C), cellulose and lignin (390–500 °C), and the weight loss over 600 °C might be responsible for the decomposition of some condensed polycyclic aromatic hydrocarbons [27,28]. Interestingly, the carbon contents in BC significantly increased as the pyrolysis temperatures increased. On the other hand, the relative percentage of the H and O content decreased. A previous study reported that the stability of the BC depends on the H/C and O/C ratios [12]. According to the European Biochar Certificate (EBC), the BC having an H/C ratio of ≤0.7 and O/C ratio of ~0.4 are very stable and could be retained in the ground soil for over 1000 years [11,12]. In the current study, we were able to produce significantly stable BC from SFSS and PNS, which have average O/C ratios of 0.21 and 0.11, respectively. It can be seen from Table 1 that by increasing the temperature from 300 °C to 900 °C for both SFS and PNS, the O/C ratio decreased from 0.5 to 0.2, indicating more stable BC because of the progress of carbonization reactions and higher carbon content in the produced char. According to earlier studies, at temperatures of 700 °C or higher, approximately 90% of the carbon sourced from different feedstocks is retained in BC. This increase in carbon content can be attributed to the transformation of carbon into well-organized layers, a process known as graphitization. Simultaneously, as the pyrolysis temperature rises, there is a noticeable reduction in the levels of hydrogen and oxygen present in the BC. This, in turn, leads to lower molar ratios of H/C and O/C, indicating the dehydration and removal of oxygen from the biomass [26]. In addition, the N-content seemed to increase at elevated temperatures, which might be due to the formation of the pyridinic-N and graphitic-N [29,30]. It can also be noticed that the N-content in PNS900 (1.2%) is higher than that of SFS900 (0.8%), which should have an effect on the adsorption analysis. Interestingly, the BET analysis indicated that the surface area of the PNS900 is considerably lower (2.1 m^2^/g) than SFS900 (85.7 m^2^/g). As reported in an earlier study, peanut shells show thermoplastic properties such as particle fusion, melting, and swelling due to the presence of a high content of condensed volatile matter, which might block the micropores of the produced PNS900 [31]. Therefore, the adsorption efficiency of the PNS900 might be dependent on other parameters such as the C-content, N-content, or other chemical interactions instead of physisorption.

### 2.2. Biochar Characterizations

#### 2.2.1. FT-IR Analysis

**Figure 1:**
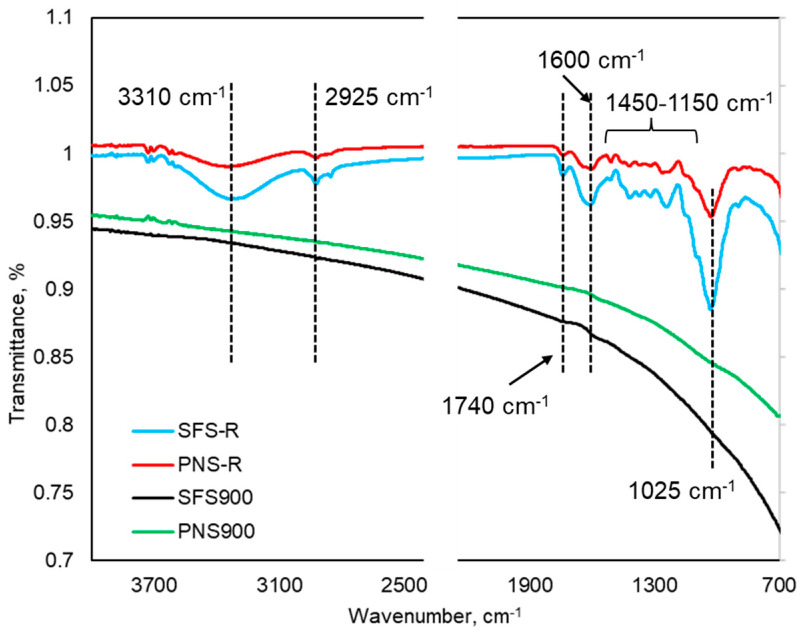
FTIR spectra of SFS R, PNS R, SFS900, and PNS900.

The structural elucidation of the raw sunflower seed shells, peanut shells (SFS-R and PNS-R), and their produced BC at 900 °C were analyzed using FT-IR and illustrated in Figure 1. As observed, different functional groups in both SFS-R and PNS-R are clearly visible from the spectra. The spectra also indicated that the SFS-R and PNS-R share similar structural units. Specifically, the broad peak that appeared at 3310 cm^−1^ is attributed to the phenolic and aliphatic hydroxyl groups [32,33]. The two adjacent peaks at 2925 and 2880 cm^−1^ are ascribed to the asymmetric and symmetric C-H stretching of aliphatic side chains, respectively [33]. The sharp peak at 1740 cm^−1^ is the characteristic peak for carboxyl groups (C=O). Another sharp peak at 1600 cm^−1^ is attributed to the C=C stretching in the aromatic ring and the C=O stretching of conjugated carbonyl groups (ketones and quinones) [33]. The aromatic guaiacyl units (from lignin) appear to be intact (1450–1150 cm^−1^) in both raw biomass [34]. The characteristic peak at 1025 is ascribed to the vibration of C-O-C [32]. By comparing the FTIR spectra of the SFS-R, PNS-R, and their biochar, it can be observed that a noticeable structural deformation happened, including the degradation of the oxygenated functional groups (hydroxyl and carboxylic groups) in the BC samples. The absence of these functional groups indicates the formation of C=C and C-H aromatic bonds, suggesting the formation of more graphite-like structure [33,35]. However, a small broader peak region can be observed at 1600 cm^−1^ which can be the remaining carbonyl structure that might be generated from the quinone and lactone structures.

#### 2.2.2. XPS Analysis

**Figure 2:**
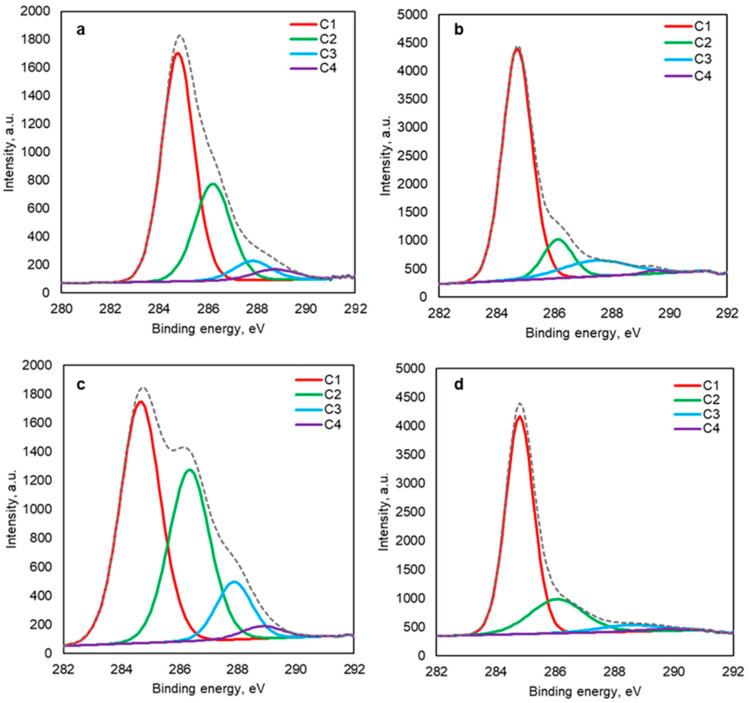
XPS carbon scans of (**a**) SFS-R, (**b**) SFS900, (**c**) PNS-R, and (**d**) PNS900.

XPS analysis was performed to study the structural changes of the SFS-R and PNS-R. Their produced biochar (SFS900 and PNS900) and their C1s scans are presented in Figure 2. Table 2 describes the relative mass concentrations of the different bond energies before and after the carbonization of the RM. In Figure 2, the C1, C2, C3, and C4 are assigned to the specific carbon bonds such as the C-C/C-H, C-OH, C=O (carbonyl), and C=O (carboxylic), respectively [28,36]. Relative mass concentrations of the C1 bond types (C-C/C-H) increased by about 13.8 and 22.6% in the BC samples in both SFS900 and PNS900, respectively, presumably due to the formation of the condensed polyaromatic structures during high-temperature pyrolysis. On the other hand, the C2 bonds (C-OH) decreased by about 16.5 and 14.7%, respectively, which indicates the decomposition of the hydroxyl groups, which can be noticed from the FTIR spectra (Figure 1). Moreover, the increasing C3 bonds (C=O, carbonyl) in SFS900 can be attributed to the formation and increasing quinone and lactone structures in the BC surfaces. However, the C3 bond types increased by around 5.8% in the PNS900 samples. The C4 bond types (C=O, carboxyl) decreased significantly in both char samples due to the decarboxylation during thermal decomposition.

#### 2.2.3. XRD Analysis

**Figure 3:**
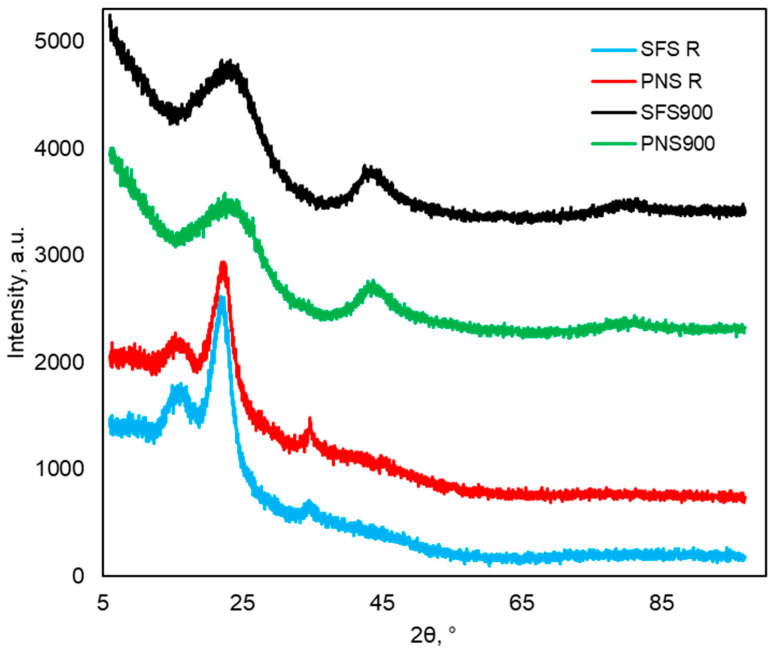
XRD spectra of SFSS, PS, SF900, PS900.

The XRD patterns of the SFS, PNS, and the produced BC at 900 °C (SFS900 and PNS900) are presented in Figure 3. As observed, the sharp peaks at 2*θ* values of around 16.4°, 22.5°, and 34.5° in the RM were due to the presence of the crystalline cellulose in the biomass samples [37]. The intensity of these peaks in SFS900 and PNS900 was reduced, indicating the decomposition of the crystalline cellulose structure under the high pyrolysis temperatures and the transformation of the biomass into amorphous materials. It can also be noticed from the XRD patterns that the diffraction angle slightly shifted to 24.3 in both BC samples, which might be due to the reducing interlayer space of the graphene that can be formed during high-temperature carbonization of biomass [38,39]. An earlier study suggested that the broad peaks appearing in the range from 20 to 30° and 40 to 50° correspond to the turbostatic form of carbon [39]. It can also be seen that the 2*θ* values of the 44.2° diffraction angle in the SFS900 and PNS900 are attributed to the structural rearrangement and the ordered graphene layers [39].

### 2.3. Adsorption Studies

#### 2.3.1. Effects of Biochar Preparation, Temperature, and Dosages on Dye Adsorption

**Figure 4:**
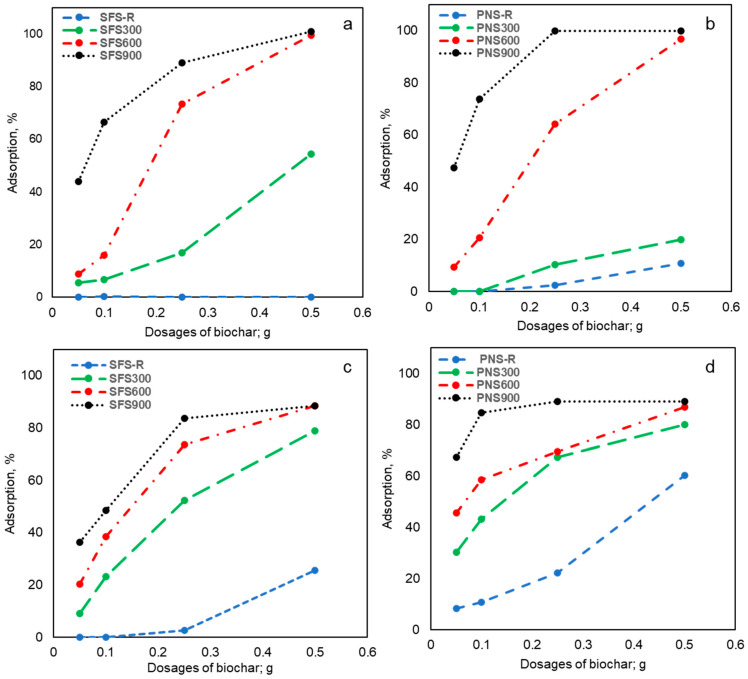
Adsorption tests of RBB and CR dyes at pH 6.5 with a dye concentration of 25 ppm; (**a**) Effects of the SFS BC on RBB dye adsorption; (**b**) Effects of the PNS BC on RBB dye adsorption; (**c**) Effects of the SFS BC on CR dye adsorption; (**d**) Effects of the PNS BC on CR dye adsorption.

Figure 4 illustrates the adsorption of RBB and CR dyes on BCs produced under different pyrolysis temperatures and applied at various dosages. Figure 4a,b represents the RBB adsorption. As observed, both raw biomass samples have poorer adsorption capacity than the BC. However, the PNS-R exhibited around 11% adsorption, which might be due to the natural porous structure of the peanut shells. It can also be noticed that with the increase in the pyrolysis temperature, the RBB adsorptions tend to increase. Interestingly, the SFS900 and PNS900 showed the maximum adsorption (100%). Increasing dosages of the BC demonstrated increasing removal of the dye. The maximum adsorption was observed upon using SFS900 at a BC dosage of 0.5 g, indicating that all the dye particles were chemically absorbed on the BC surfaces. In the case of the PNS900, the maximum adsorption can be achieved at a dosage of 0.25 g, which implies that the PNS BC could be a more efficient RBB adsorber than the SFS BC.

Similarly, the CR dye adsorption by the raw biomass and their produced char also showed an upward trend as the pyrolysis temperature increased. However, the adsorption efficiency was lower (~89%) than RBB (100%). Moreover, earlier studies also reported the pH effects on CR dye adsorptions, showing that above the pH of 5.5, the CR behaves more anionic in nature, which might decrease the adsorption as the BC has some negatively charged functional groups such as quinone, carboxylic groups, etc. [40,41]. Another factor that causes the CR dye to be less effective is the chemical nature of the dyes. The RBB is an anthraquinone dye; on the other hand, the CR is an azo dye. The RBB is an anthraquinone dye with more negative interactive functional groups (quinones, sulfonyl groups) that can form H-bonds during adsorption. On the other hand, the CR is an azo with no functional groups to interact with BC, resulting in less adsorption and less dye removal. Another reason for the higher adsorption capacity of the PNS900 could be explained by the total nitrogen content in BC (Table 1). The increasing nitrogen content in the PNS900 might be due to the formation of the pyridinic-N and graphitic-N, which are positively charged and can interact more with the negatively charged dye molecules [29]. However, increasing BC dosages increased the adsorption due to an increase in the total adsorbent surface areas and the number of available adsorption sites. Interestingly, the SFS-R and PNS-R are also able to remove around 26% and 60% of CR at 0.5 g dosages, which might be due to the presence of available oxygenated functional groups such as the hydroxyl groups and the carboxylic groups of the biomass, which are mainly coming from the cellulose and lignin. The presence of these functional groups can be co-related to the XPS results (Figure 2). Overall, it can be observed from Figure 4 that PNS900 demonstrated higher adsorption of both dyes compared to SFS900, which can also be explained in Table 1. As we can see, the carbon content of PNS900 (H/C 0.42, O/C 0.11) is better than the SFS900 (H/C 0.9, O/C 0.2), indicating that PNS900 is more stable with graphite-like structures (Figure 3) and contains more polyaromatic structures (Table 2, Section 2.2.2).

#### 2.3.2. Effects of Contact Time on Adsorption

**Figure 5:**
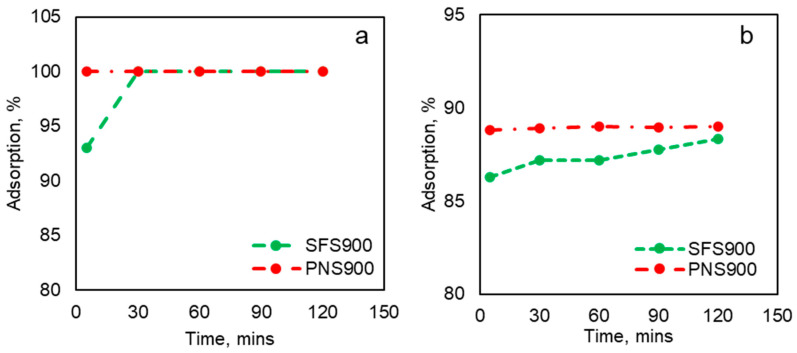
Effects of adsorption on contact time at pH 6.5 with a BC dosage of 0.5 g. (**a**) RBB dye adsorption; (**b**) CR dye adsorption.

The effects of the contact time on the RBB and CR adsorption were also studied in the current work and reported in Figure 5a,b. Figure 5a represents the adsorption of RBB by SFS900 and PNS900 at 5, 30, 60, 90, and 120 min. The study showed that PNS900 is more efficient than SFS900. The PNS900 exhibited rapid adsorption and reached equilibrium in less than 5 min of contact time. On the other hand, SFS900 took around 30 min to reach equilibrium. A similar trend can also be noticed while CR was adsorbed by the same samples at equal contact times (Figure 5b). This might be due to external and liquid film diffusion of the dye molecules in the BC in the first stage (t = 1–10 min). In this stage, the dye molecules from the bulk solution penetrate and are adsorbed into the external surface of the BC [42]. However, in the case of SFS900, the equilibrium stage achieved after 30 min indicates the intermediate stage (t = 20–120 min). In this stage, intra-particle diffusion occurs, and the dye molecules enter into the micro and macropores of the BC from the exterior surfaces before the adsorption reaches equilibrium [42]. Moreover, as the contact time increases, dye molecules tend to group together, preventing them from penetrating deeper into the adsorbent structure to more energetically favorable sites. This clustering effect diminishes the significance of the contact time as the pores become saturated and begin to obstruct the diffusion of these clustered dye molecules within the adsorbent material [43].

### 2.4. Adsorption Mechanism of the Dyes

The process of adsorption is influenced by factors such as the chemical structures of the dye molecules, the types of functional groups present in them, the surface characteristics of the BC, and the interactions between the dye molecules and the BC. The binding of dye molecules to the adsorbent can occur through multiple competing physical adsorption (physisorption) or chemical adsorption (chemisorption) mechanisms, and the dominant adsorption mechanism is highly dependent on the specific interaction between the surface of the adsorbent and the adsorbate. In many instances, the accumulation of dye molecules on agricultural biomass-derived BC results from a combination of different interactions, such as physical interactions, electrostatic interactions, and hydrogen bonding, all of which can occur during adsorption [20]. The primary mode of interaction between the aromatic rings of the carbonaceous material and anionic dye molecules is proposed to be chemisorption, primarily involving strong π–π stacking and anion–cation interactions [20].

**Figure 6:**
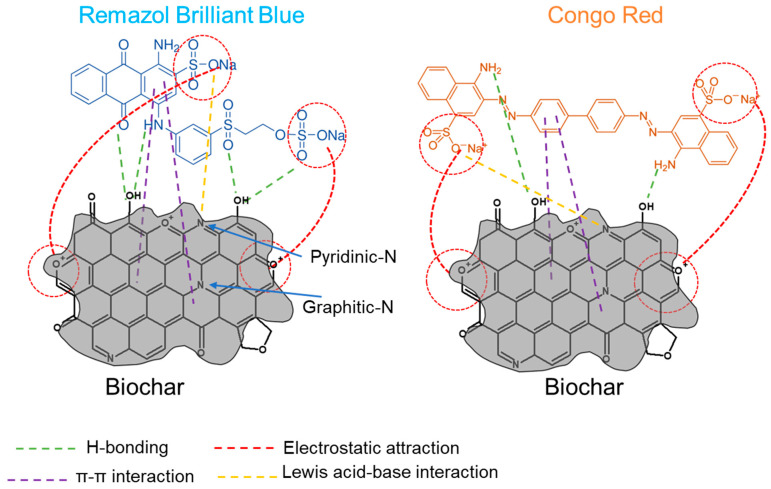
Possible adsorption mechanism of the biochar.

As seen from Table 1, the BET surface area of SFS900 is significantly higher (85.7 m^2^/g) than PNS900 (2.1 m^2^/g), which indicates that the adsorption efficiency of the PNS900 did not depend on the surface area. In this context, RBB is an anionic anthraquinone dye with two sulfonic groups in its molecular structure, ionizing in aqueous solutions and forming colored anions in conjunction with aromatic rings. The quantity of anions (−SO_3_^−^) plays a significant role in the adsorption of RBB. Moreover, it has an anthraquinone group and a sulfonyl group, which can participate in forming more H-bonds with the BC molecules (Figure 6). On the other hand, CR is an anionic azo dye that has sulfonic groups similar to RBB. However, there are no sulfonyl groups or quinone groups in CR; hence, the formation of H-bonds is less on the BC surface, resulting in lower adsorption. This could be the possible reason for the lower adsorption of CR than that of the RBB. However, BC derived at higher pyrolysis temperature will have increased aromaticity due to the formation of condensed polycyclic aromatic compounds, which may lead to an increase in the π–π and hydrophobic interaction [20,44,45]. As the preparation temperature rises, there is a decline in the quantity of oxygen-containing functional groups (specifically, the -COH and -COOH groups, discussed in Section 2.2.2.), leading to a reduction in the extent of deprotonation of these groups. Consequently, the negative surface charge diminishes, which results in higher adsorption of the anionic dyes due to less electrostatic repulsion. Additionally, at elevated pyrolysis temperatures (700–900 °C), the biochar should have less negatively charged carboxylate groups (Table 2); however, positively charged oxonium groups may form [46]. Due to electrostatic attraction, these oxonium groups can actively interact with the negatively charged sulphonic groups (acidic to neutral pH). Therefore, regardless of the dye molecules, the adsorption of the dyes is higher in SFS900 and PNS900 compared to the BC produced at other lower temperatures. The Lewis acid-base interaction might be another possible adsorption mechanism. Higher temperatures (>600 °C) may lead to the formation of pyridinic-N in the biochar surfaces, which can donate electrons and act as a Lewis base. Reversely, the electron withdrawal properties of nitrogen may lead to the formation of the Lewis acid [29]. Moreover, interactions involving π–π electron donor and acceptor (EDA) play a crucial role in the adsorption process of planar aromatic pollutants onto biochar surfaces resembling graphene. Earlier studies postulated that when graphitization is incomplete, there is an irregular distribution of charge among the aromatic rings in biochar, leading to alterations in the electron density [29]. This results in the formation of either π-electron-rich or π-electron-deficient regions within the biochar structure. These regions, characterized by excess or insufficient electrons, can engage in electron exchange with aromatic contaminants through π–π EDA interactions. The presence of graphitic-N has the potential to enhance these interactions. Notably, studies by Wang et al. demonstrated that nitrogen functional groups withdraw π-electrons from the graphene layer, establishing positive environments. These positive surroundings may act as π-electron acceptors, facilitating interactions with π-electron-enriched aromatic rings, such as those found in acid red 18, an azo dye containing sulphonic groups [29,47]. In this context, the presence of the higher nitrogen content (Table 1) and the detected graphite-like structures (Figure 3) in the SFS900 and the PNS900 can be correlated with higher adsorption.

## 3. Comparison of Recent Studies on Dye Removal

Table 3 presents a comparison of recent studies that investigated the removal of dye using biochar derived mainly from food wastes. In one study, walnut shells were carbonized for the removal of toxic RBB at pH 2 [20]. They carried out chemical activation using CaCl_2_, HNO_3_, and KOH. Nitric acid-activated char exhibited superior performance (87.3% removal efficiency) due to increased surface area and pore volume. Another study focused on nutmeg shell biochar removing RBB dye at pH 12, which demonstrated maximum adsorption after 110 min [48]. Similarly, activated jackfruit peel biochar showed effective dye removal at pH four within 420 min. Raj et al. investigated RBB dye adsorption using sewage sludge-derived biochar, achieving rapid efficiency within 20 min [49]. Green pea peel-derived biochar exhibited 40% dye removal within 90 min at neutral pH for CR dye [22]. Comparing these studies with our findings, PNS900 biochar demonstrated rapid adsorption (100% in less than 30 min) for RBB dye removal at neutral pH, eliminating the need for additional neutralization steps.

## 4. Materials and Methods

### 4.1. Materials

Whole roasted sunflower seeds and peanuts were purchased from the local market, and their shells were separated for biochar production. Remazol Brilliant Blue (RBB) and Congo Red (CR) dyes were purchased from Sigma-Aldrich (Burlington, MA, USA) and used without further purification. Nitrogen gas was supplied by Linde Canada (Mississauga, ON, Canada).

### 4.2. Pretreatment of the Raw Materials and Biochar Production

The raw sunflower seed shells (SFS-R) and peanut shells (PNS-R) were meticulously cleaned by washing them multiple times with deionized (DI) water to eliminate any impurities. Subsequently, they were subjected to a drying process in an oven at 105 °C for 48 h prior to pyrolysis.

The dried SFS-R and PNS-R were subjected to pyrolysis within a tubular furnace while being continuously exposed to a flow of nitrogen gas. Briefly, 4 g (dry basis) of slightly ground dry biomass was inserted into the tube furnace (Across International-STF1200, Livingston, NJ, USA) in each experiment and allowed to combust in the absence of oxygen at three different temperatures: 300, 600, and 900 °C. This process was carried out with a nitrogen flow rate of 250 mL/min and a heating rate of 10 °C/min, lasting for 3 h. After cooling down, the produced biochar was collected and washed with DI water to eliminate soluble salts. Subsequently, the biochar was dried in an oven at 105 °C, and the yields were measured.

### 4.3. Characterizations of the Biochar

#### 4.3.1. Elemental Analysis

The CHNS of the SFS-R and PNS-R and their char were measured using an Elemental analyzer (Vario EL Cube, Lagenselbold, Germany).

#### 4.3.2. Fourier-Transform Infrared Spectroscopy (FT-IR)

The structural changes of the SFS-R, PNS-R, and their char were investigated using a Bruker Tensor 37 FT-IR instrument (Bruker, Karlsruhe, Germany), which was equipped with a PIKE MIRacle Diamond Attenuated Total Reflectance (ATR) accessory. Around 1.5–2 mg of dried powdered samples were placed on the ATR crystal of that instrument, and we measured the transmittance (a.u.) [52]. For all samples, 64 scans were conducted between the wavenumber of 4000–500 cm^−1^. The resolution of the spectra was 4 cm^−1^.

#### 4.3.3. X-ray Photoelectron Spectroscopy (XPS)

XPS analyses of the SFS-R, PNS-R, and their char were conducted using a Kratos AXIS Supra, Kyoto, Japan, with the monochromatic AL anode. A previously described method was followed to analyze the samples [28]. The dried samples were analyzed using a 375 W X-ray. In this experiment, the number of steps, sweep, and dwell time were 230, 60 s, and 260 ms, respectively. Data analysis was performed using the ESCApe software (V1.2.0.1325).

#### 4.3.4. X-ray Diffraction (XRD) Analysis

The crystalline nature and crystal type of the SFS, PNS, and their char were identified by XRD (X’Pert Pro X-ray diffractometer, PANalytical, Almelo, The Netherlands) with a PIXcel detector and Kα radiation (λ = 1.54 Å). The X-ray diffractograms were acquired with a current of 40 mA and a voltage of 45 kV using a copper X-ray tube with a range of 6–40° at a scan speed of 0.005 deg s^−1^.

#### 4.3.5. Brunauer, Emmett, and Teller (BET) Analysis

The BET surface area was assessed through nitrogen (N_2_) adsorption isotherm and desorption at 77 K (liquid nitrogen temperature). Nitrogen adsorption–desorption isotherms were obtained at the boiling point of liquid nitrogen using Quantachrome NOVA 2200E, Boynton Beach, FL, USA. Prior to testing, the sample underwent a 10-h degassing process at 120 °C under vacuum.

### 4.4. Adsorption Tests

Adsorption analysis was conducted using a UV-vis spectrometer (Varian Cary 50 Scan UV Visible Spectrophotometer, Santa Clara, CA, USA). In this analysis, 25 ppm solutions of each dye were employed. The absorbance of the RBB and CR dyes was measured at wavelengths of 593 nm and 497 nm, respectively. To summarize the procedure, specific quantities of biochar were introduced into 30 mL of the dye solution within a 50 mL falcon tube. Subsequently, the tubes were agitated for 120 min at room temperature in a shaker (Excella E5 platform shaker, Edison, NJ, USA). The mixtures were then filtered through a 0.45 µm cellulose acetate-based syringe filter (Sartorius, Gottingen, Germany), and the resulting supernatants were used for adsorption analysis. The remaining dye concentration in the solutions was determined using pre-established calibration curves for each dye. All the adsorption tests were performed in triplets, and the average was reported. Equation (1) was used to calculate the percentage of the adsorption for each dye, where C_0_ and C_f_ are the initial and final (after adsorption) concentrations of the dye solutions.

Adsorption, % = {(C_0_ − C_f_)/C_0_} × 100 (1)

## 5. Conclusions

The current study underscores the significance of utilizing food waste as a valuable biomass source. By directly employing sunflower seed shells (SFS) and peanut shells (PNS) through pyrolysis, this research successfully generated biochar capable of efficiently removing RBB and CR dyes from wastewater. Notably, biochars produced at 900 °C exhibited enhanced environmental stability, particularly PNS900, attributed to its higher carbon content (83%) and confirmed graphene structures via XRD analysis. The XPS analysis unveiled increased C-C/C=C bonds, indicating the formation of polyaromatic moieties and the degradation of oxygenated functional groups (C=O/COOH). As anticipated, biochars produced at 900 °C demonstrated superior dye removal, with PNS900 outperforming SFS900. Specifically, RBB dye removal was approximately 11% higher with PNS900 at similar dosages compared to SFS900, potentially due to its higher carbon content (83.7%). Despite both RBB and CR being anionic dyes, chemical functional groups such as anthraquinone and azo groups likely played active roles in biochar adsorption by electrostatic attraction. Moreover, the high carbon content and the increased nitrogen content (1.2%) in PNS900 indicated the formation of the organic N-containing functional groups (pyridinic-N and graphitic-N), which can facilitate the Lewis acid-base and π–π EDA interactions with the anionic dyes. The findings of this study offer valuable insights into converting food waste into biomass for producing biochar essential for water decontamination processes, serving as a significant reference for future research endeavors in this field.

## Figures and Tables

**Table 1:** Yield and elemental characterization of the raw and BC samples.

Raw Materials	Temperature, °C	Yield, %	N, %	C, %	H, %	H/C, Molar Ratio	O, %	O/C, Molar Ratio	BET Surface Area, m^2^/g
SFS	Raw	NA	0.4	41.8	7.1	2.0	50.5	0.9	ND
	300	49.2	0.9	56.7	6.9	1.4	35.3	0.5	7.2
	600	36.7	0.7	64.1	5.7	1.0	29.3	0.3	ND
	900	30.2	0.8	72.3	5.5	0.9	21.2	0.2	85.7
PNS	Raw	NA	0.4	47.7	2.3	0.6	49.4	0.8	ND
	300	56.1	ND	ND	ND	ND	ND	ND	ND
	600	32.4	ND	ND	ND	ND	ND	ND	ND
	900	30.2	1.2	83.7	2.9	0.42	12	0.11	2.1

NA = Not Applicable; ND = Not Determined.

**Table 2:** Binding energies and mass concentrations of carbon and oxygen species of the SFS-R, PNS-R, SF900, and PS900.

		Mass Concentration, %			
Bond Types	Assignments	SFS-R	PNS-R	SFS900	PNS900
C1	C-H, C-C, C=C	60.9	50.2	74.7	72.8
C2	C-OH/C-O-C	29.1	36.5	12.6	21.8
C3	C=O, carbonyl	6.1	10.7	12	4.9
C4	C=O, carboxylic	3.9	2.7	0.7	1.0

**Table 3:** Recent studies on dye removal by various biomass-derived biochar.

Biomass	Pyrolysis Condition Temperature	Dyes	Dye Concentration, mg/L	Biochar Dosages, g/L	Max. Removal Efficiency	Contact Time, min	pH	References
Walnut	700	RBB	200	5	65	720	2	[20]
Swege sludge	450	RBB	30	100	67	20	10	[49]
Orange Peels	800	MB	10	10	99	30	7	[50]
Jack fruit peel (Activated)	550	RBB	25	1	90	420	4	[51]
Nutmeg seed shells	500	RBB	52.5	3	93.7	110	12	[48]
Green pea peels	700	CR	100	1	40	90	7	[22]
Peanut shell	900	RBB	25	8.3	100	30	6.5	Current study

## Data Availability

Data are contained within the article.
